# Hospitalization and Care Trajectories Near End of Life Among Older Adults – A Nationwide Cohort Study

**DOI:** 10.1093/geroni/igaf122.4131

**Published:** 2025-12-31

**Authors:** Naemi Herzog, Rahel Laager, Philipp Schütz, Beat Müller, Alexander Kutz

**Affiliations:** University of Basel / Cantonal Hospital Aarau, Zurich, Switzerland; Children’s Hospital Lucern, Lucern, Luzern, Switzerland; Cantonal Hospital Aarau, Aarau, Aargau, Switzerland; Cantonal Hospital Aarau, Aarau, Aargau, Switzerland; Cantonal Hospital Aarau, Aarau, Aargau, Switzerland

## Abstract

The final year of life accounts for a disproportionate share of health-care utilization. Characterizing hospitalization patterns, causes, and interventions during this period, and examining how these trajectories differ by frailty, is crucial for anticipating needs and improving end-of-life care. In this retrospective cohort study using Swiss national administrative claims of adults aged ≥65 years (2013-2022), we assessed temporal trends in hospitalizations within 1 year of death. The main outcome included causes of hospitalizations and number of invasive care interventions, stratified by frailty. Among 186,349 older adults, causes of hospitalization and interventions remained largely stable over time, with cardiac disease, cancer and injury-related conditions predominating. In frailer individuals, psychiatric and neurological conditions were more frequent in addition to injuries and cardiac diseases. Overall, 51.1% of individuals were hospitalized within the final three months of life. Among less frail individuals, cancer-related hospitalizations increased substantially (8.7% at 12 months before death to 26.6% in the last month of life, standardized mean difference [SMD] −0.482, p < 0.001), whereas in frailer individuals, the absolute increase was smaller (1.4% to 6.1%, SMD -0.252, p < 0.001), alongside increases observed for cardiac (10.1% to 18.3%, SMD -0.238 p < 0.001) and infectious diseases (9.5% to 15.7%, SMD -0.189 p < 0.001). Intervention rates increased modestly near death (39.4% to 42.0%, SMD −0.053, p = 0.005). In conclusion, hospitalization trajectories in the last year of life differ by frailty, with cancer dominating in the less frail and non-cancer causes in the frail. Tailored approaches are needed to improve alignment of end-of-life care.

